# Next-generation Approaches in Targeting Polycystic Ovarian Syndrome: Innovative Strategies

**DOI:** 10.2174/0109298673368951250404170052

**Published:** 2025-05-12

**Authors:** Pavithra Lakshmi Narayanan, Subalakshmi Sugumar, Rapuru Rushendran, Chitra Vellapandian

**Affiliations:** 1 Department of Pharmacology, SRM College of Pharmacy, SRM Institute of Science and Technology, Kattankulathur, 603203, Chengalpattu District, Tamil Nadu, India

**Keywords:** Bioinformatics, innovative research, machine learning, polycystic ovarian syndrome, therapeutic strategies, molecular docking

## Abstract

Polycystic Ovary Syndrome (PCOS) is a complex endocrine disorder that affects millions of women worldwide and is characterized by ovarian dysfunction, hyperandrogenism, and metabolic abnormalities. The traditional diagnostic and therapeutic approaches often fail to address the multifaceted nature of PCOS. Recent advancements in next-generation sequencing (NGS), bioinformatics, and precision medicine have paved the way for innovative research and therapeutic strategies that promise to revolutionize PCOS management. This review focuses on exploring the genetic and molecular mechanisms of PCOS using innovative methodologies, such as genome-wide association studies (GWAS), transcriptomics, and computational approaches. Integrating big data analytics and machine learning algorithms enhances the predictive accuracy of PCOS diagnoses and treatment outcomes. In addition, the emergence of personalized medicine has enabled tailored therapeutic interventions based on individual genetic profiles and phenotypic expression. Furthermore, we explored the development of novel pharmacological agents and combinational therapies to enhance the understanding of PCOS pathophysiology. These approaches also focus on reducing inflammation, improving insulin sensitivity, and optimizing hormonal balance to achieve optimal health outcomes. The potential of digital health tools, including mobile applications and wearable technologies, to support self-monitoring and patient engagement in PCOS management is also highlighted. In conclusion, the integration of next-generation technologies and innovative research is necessary to transform the field of PCOS diagnosis and treatment, offering hope for more effective and individualized care. These underscore the importance of continued investment in advanced research methodologies and the adoption of personalized therapeutic strategies to address the complexities of PCOS.

## 1 INTRODUCTION

**Fig. (1):**
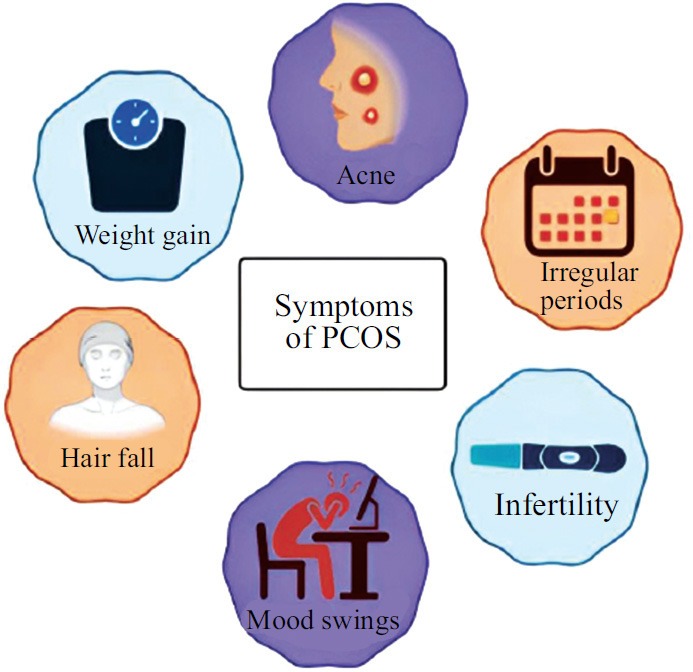
Illustration depicting the major symptoms of PCOS which affects the self-esteem and confidence of the affected women in the entire population.

Polycystic ovary syndrome (PCOS) is a major public health issue and one of the most prevalent hormonal disturbances affecting women of reproductive age. PCOS is a hyperandrogenic disorder characterized by chronic oligo-anovulation and polycystic ovarian morphology [1]. This condition affects an estimated 8-13% of women of reproductive age, with up to 70% of cases remaining undiagnosed [2, 3]. In India, PCOS affects one in five women [4]. According to the NIH diagnostic criteria, the prevalence of PCOS in the US, UK, Spain, Greece, Australia, Asia, and Mexico increased from 6% to 9%. Obesity and overweight are linked to PCOS where observational data shows that 33-88% of women with PCOS are overweight or obese and about 75% of PCOS patients develop insulin resistance [5]. Furthermore, a comprehensive survey conducted across India in 2020 indicated that approximately 16% of women aged 20 to 29 years are afflicted with PCOS [6, 7]. PCOS was first described by Stein and Leventhal as a syndrome characterized by oligo-amenorrhea and is therefore also known as Stein-Leventhal Syndrome [8]. It is commonly associated with hirsutism, acne, and obesity with psychological impairments, including depression and other mood disorders, as well as metabolic abnormalities, primarily insulin resistance and compensatory hyperinsulinemia (Fig. **1**). These metabolic disturbances are recognized as significant contributors to altered androgen production and metabolism.

The precise cause of PCOS is currently unknown, but there are associations with insulin resistance, low-grade inflammation, and genetic factors. Insulin, a hormone produced by the pancreas, regulates blood sugar levels by facilitating the movement of glucose from the blood into cells, where it is metabolized for energy. Insulin resistance occurs when body tissues do not respond effectively to insulin, necessitating increased insulin production. This excess insulin can stimulate the ovaries to produce higher levels of testosterone, disrupting follicle development and inhibiting normal ovulation [9, 10]. Hormonal imbalances are also significant contributors to PCOS. Elevated levels of testosterone, luteinizing hormone (LH), and in rare cases, prolactin, along with decreased levels of sex hormone-binding globulin (SHBG), can lead to the development of PCOS [11, 12]. Genetic predisposition may also play a role. A familial history of PCOS, such as in a mother, sister, or aunt, increases the likelihood of developing the condition, indicating a potential genetic component [13]. However, no specific gene linked directly to PCOS has been identified. Thyroid conditions, notably hypothyroidism, and Hashimoto's thyroiditis, have been related to PCOS. Hypothyroidism can lead to the development of polycystic ovarian morphology, which affects ovulation and hormone balance [14].

The pathogenesis of PCOS began to be understood with a 1958 report that found elevated urinary luteinizing hormone (LH) levels in the four cases studied [15]. Existing studies on the pathogenesis of PCOS have limitations, particularly when considering its multifactorial etiology, including ethnic and regional factors. Being born small for gestational age (SGA) or being the child of a hyperandrogenic mother might be considered clinical markers for developmental programming related to steroids. However, individuals not exposed to excess steroids and offspring of non-hyperandrogenic mothers can also develop PCOS [16]. This suggests that both postnatal environmental factors and genetic predispositions may contribute to the origin of this disorder. Healthcare providers typically assess three primary characteristics before diagnosing PCOS: anovulation, elevated androgen levels, and ovarian cysts [17]. The presence of one of these features may indicate PCOS. Due to the lack of comprehensive understanding of PCOS, various expert groups use different criteria for diagnosis.

Generally, they consider menstrual irregularities, high androgen levels, and the presence of multiple cysts of a specific size on one or both ovaries as detected by ultrasound. PCOS does not have a specific cure and is typically managed based on its symptoms, resulting in varied treatment approaches. Major treatments include planning a proper diet and weight loss, which can improve the condition. Irregular or absent menstruation and fertility-related issues can be treated with medication. Anti-androgens may be used to address excessive hair growth. A few other classes of drugs like oral contraceptives, ovulating agents, anti-diabetic drugs, anti-inflammatory agents, and a few nutritional supplements are administered for combating the symptoms of PCOS. For fertility problems associated with PCOS that do not respond to medication, laparoscopic ovarian drilling may be an option [18]. Synthetic medications have demonstrated significant efficacy in managing PCOS. However, their prolonged use is complicated by adverse drug reactions. In contrast, regular herbal use is deemed safer and more effective for treatment [19]. Due to higher recovery rates and patient acceptance, individuals increasingly rely on herbal therapies as alternatives to synthetic medications for controlling and treating PCOS [20].

The research in the field of PCOS remains critical in the current scenario as a major population of women is getting affected by the disorder. Since the exact pathophysiology of PCOS remains unknown, the utilization of recent techniques like computational methods and AI tools helps in the determination of the optimal target and scrutinization of the potential lead [21, 22]. One significant gap lies in the multifaceted nature of PCOS, fails to capture the traditional diagnostic and therapeutic methods. The Rotterdam, NIH, and Androgen Excess Society guidelines are among the commonly used diagnostic criteria that place a strong emphasis on phenotypic signs such as polycystic ovarian morphology, hyperandrogenism, and ovulatory dysfunction. These standards, however, frequently ignore the variety of PCOS, especially its metabolic and psychological aspects, which are essential for comprehensive care. Additionally, new research indicates that biomarkers including gut microbiome changes, inflammatory markers, and anti-Müllerian hormone (AMH) may offer more information about the complexity of disease, although they are still not widely used in clinical practice. On the therapeutic front, the reliance on generic interventions, such as oral contraceptives, insulin sensitizers, and lifestyle modifications, fails to account for the diverse phenotypic and genetic profiles of patients. These one-size-fits-all approaches often yield suboptimal outcomes and do not address the underlying pathophysiology of PCOS. Moreover, the lack of integration between therapeutic modalities, such as pharmacological treatments, dietary interventions, and mental health support, further limits the effectiveness of current strategies. To tackle this, researchers are leveraging next-generation technologies such as genome-wide association studies (GWAS), transcriptomics, and computational approaches to better understand the genetic and molecular mechanisms underlying the condition. Another gap is the limited predictive accuracy of current diagnostic models and treatment outcomes, which is being addressed through the integration of big data analytics and machine learning algorithms to improve precision. Additionally, there is a lack of personalized therapeutic options, which researchers are overcoming by developing tailored interventions based on individual genetic profiles and phenotypic expressions [23, 24]. The absence of comprehensive and effective pharmacological solutions is being tackled through the exploration of novel drugs and combinational therapies aimed at reducing inflammation, enhancing insulin sensitivity, and optimizing hormonal balance. Finally, while digital health tools have the potential to transform PCOS management, their widespread adoption faces challenges related to user engagement and accessibility, which researchers are attempting to resolve by designing more intuitive and inclusive technologies. These efforts collectively aim to bridge existing knowledge gaps and pave the way for more effective and individualized approaches to PCOS care. Thus, the current piece of work majorly discusses recent innovations in the field of medicine for the diagnosis and monitoring of PCOS conditions followed by the discussion on next-generation research through a series of computational techniques which stay as a strong basement for the drug discovery process.

## 2 INNOVATIVE DIAGNOSTIC TOOLS FOR PCOS

In recent years, the integration of artificial intelligence (AI) and machine learning (ML) in medical diagnostics has enabled physicians to personalize treatment plans for patients with PCOS, considering individual patient characteristics, such as age, weight, and medical history [25-27]. This personalized approach has shown promising results in improving the overall health and well-being of patients with PCOS, as well as reducing the risk of long-term health complications associated with the condition [28]. Additionally, with the use of newly developed PCOS tracker applications, women can proactively monitor their lifestyle patterns and symptoms related to PCOS, leading to better management of the condition even before being diagnosed by physicians [29]. Furthermore, these applications allow women to track their menstrual cycles, blood sugar levels, and insulin resistance, which can help them identify the early signs of PCOS and seek medical advice promptly. The list of innovative diagnostic tools that can significantly benefit patients with PCOS is discussed in Table **1**.

## 3 *IN SILICO* GENOMIC AND MOLECULAR RESEARCH

### 3.1 Gene Sequencing and Identification

Gene sequencing in polycystic ovary syndrome (PCOS) is crucial for understanding the genetic basis of the disorder. By identifying specific genetic variants associated with PCOS, researchers can uncover the underlying molecular mechanisms that contribute to its development. The Genetic underpinnings of polycystic ovarian syndrome (PCOS) were performed by focusing on single nucleotide polymorphisms (SNPs). Genome-wide association studies (GWAS) were used to identify relevant genes various bioinformatics tools were employed to analyze the SNPs. The methodology involves collecting DNA samples, genotyping single nucleotide polymorphisms (SNPs), and comparing allele frequencies between cases and controls. Statistical tests identify significant associations, often visualized as Manhattan plots. The results elucidate genetic risk factors and probable biochemical mechanisms associated with the illness. The study identified 25 PCOS-related genes and conducted pathway analysis to understand their biological roles. A total of 16,71,896 SNPs were initially identified, which were then filtered to focus on missense variants and other functional SNPs. Seven deleterious SNPs were highlighted using six different pathogenicity prediction tools. The identified deleterious SNPs in genes such as ERBB4, GATA4, INSR, LHCGR, SUOX, and YAP1 were discussed for their potential roles in disease progression. The study also highlighted the impact of these SNPs on protein stability and function, as well as their regulatory roles through miRNA binding and transcription factor binding sites. The findings suggest that these genetic variations could significantly influence the pathophysiology of PCOS and contribute to its heritability [30]. Sarkar *et al. *[31] emphasize the importance of understanding the regulatory networks controlled by transcription factor binding sites (TFBS) in PCOS. Using *in silico* tools, the study identified four over-represented TFBS like Staf, E47, CCAAT, and CRE-BP1/c-jun in genes co-expressed in ovarian theca cells of PCOS patients. Staf, a zinc finger protein known for activating RNA polymerase II and III promoters in small RNA genes has human homologs ZNF76 and ZNF143. These homologs recognize similar response elements and play roles in transcriptional activation and repression.

The over-representation of Staf binding sites in PCOS-related genes might contribute to the regulatory mechanisms of the syndrome. Similarly, the transcription factor, E47, and related bHLH factors were associated with the regulation of FSH and its receptor (FSHR), making it critical for ovarian follicular development and estrogen biosynthesis. The presence of E-box sequences in the FSHR promoter region indicated that E47 may regulate FSHR gene expression, an essential element for follicular maturation and ovulation. The CCAAT Box, its associated transcription factors, and CRE-BP1 (cAMP-Response Element Binding Protein 1) play an integral part in the regulation of genes involved in key pathways affected in PCOS, including steroidogenesis, insulin signalling, and follicular development thus providing a major impact on the PCOS functioning. These findings suggest that these TFBS might be involved in the regulatory mechanisms underlying PCOS. The findings from [32] explore the impact of single-nucleotide polymorphisms (SNPs) in the CYP11A1 gene, associated with polycystic ovary syndrome (PCOS), on the properties of the encoded proteins. On analysis of 78 SNPs, the research identified 17 significant mutations that alter the structure, sub-cellular localization, and physicochemical properties of the mutated proteins. The study by Dhar *et al. *[33] investigates the genetic basis of obesity-mediated PCOS by comparing obese and lean phenotypes using a bioinformatics-driven approach. Biological samples were collected from two participants selected based on anthropometric parameters and confirmed PCOS diagnosis. DNA extraction was followed by clinical-exome sequencing using the Next-generation Illumina platform, identifying 26,550 variants. Of these, 5,170 were common, while 2,232 and 2,322 variants were unique to lean and obese PCOS phenotypes, respectively. Key genes, including CYP1A1, CYP19A1, ESR1, AR, AMH, PTEN, and AdipoR1, were associated with leptin signalling impairment, insulin resistance, and estrogen resistance. The study highlights distinct genetic mechanisms between lean and obese PCOS, suggesting that these variations contribute to different molecular signalling pathways and disease complexity.

### 3.2 Network Pharmacology

Network Pharmacology plays a vital role in the identification of core pathways and PPI involved in the pathophysiology of the disease or disorder. As the pathophysiology of PCOS remains unknown, it is essential in the determination of core targets to process the research in drug discovery. The findings of Tiwari *et al. *[36] suggest that four prominent targets AKT1, MAPK1, MAPK3, and STAT3, were identified through PPI and network analysis with the help of tools mentioned in Table **3**. The network analysis of Begum *et al. *[37] highlighted targets like AKT1, PTGS2, PPARG, PPARA, ESR1, LDLR, GSK3B, CNR1, ACE, and ESR2 for their contributions towards PCOS. Thus, these findings give a clear picture of the important targets responsible for the potential pathophysiology of PCOS and pave the way for future research into it.

Various bioinformatics tools such as CELLO2GO, ProtParam, PHYRE2, I-Mutant, SIFT, and PolyPhen, were employed to assess these changes. The results indicated that most missense mutations negatively impacted protein stability and function, suggesting their potential as biomarkers for assessing PCOS risk in women. The research findings of [34] suggest the role of angiogenesis-related genes and their regulatory miRNAs in the pathophysiology of polycystic ovary syndrome (PCOS). Through an extensive literature search and reanalysis of ovarian gene expression datasets, the study identified angiogenesis-related genes that are differentially expressed in the ovaries of women with PCOS. These genes were then used to predict their regulating miRNAs, leading to the development of a miRNA-mRNA network. The research discovered that miR-218-5p, miR-214-3p, miR-20a-5p, and miR-140-3p were upregulated in granulosa cells of women with PCOS, targeting genes within the PI3K/Akt signalling pathway, which is vital for angiogenesis. The study conducted by Butler *et al. *[35] explored the differential expression of microRNAs (miRNAs) in the follicular fluid of 29 anovulatory women with PCOS and 30 controls undergoing *in vitro* fertilization (IVF). Among 176 detected miRNAs, 29 showed significant differences, with seven prominently linked to clinical features of PCOS such as insulin resistance (IR), inflammation, and hormonal imbalances. Correlations were observed between miR-199b-5p and AMH, miR-382-5p and free androgen index (FAI), and miR-93-3p and CRP in PCOS patients. Functional analysis revealed miRNAs involved in reproductive pathways, inflammation, and metabolic processes. The study highlighted miRNAs like miR-127-3p, miR-382-5p, and miR-425-3p correlating with fertilization rates in controls. While findings confirm miRNAs as potential regulators of ovulatory dysfunction and IR in PCOS, the inhibitory mechanisms and population differences require further research. The findings indicate that these miRNAs and their interactions with angiogenesis-related genes play a significant role in the impaired follicular angiogenesis seen in PCOS, contributing to its pathophysiology. The list of tools, online software and web servers used in the computational research for combating the symptoms of PCOS are listed in Table **2**.

### 3.3 Molecular Docking and Molecular Dynamics Simulations

Molecular docking studies involve the determining of binding affinity of the ligand with the targeted proteins and molecular dynamics studies determine the complex stability of ligands and proteins in the environment. Studies for determination of potential phytoconstituent was majorly carried out in a series of natural herbs as they act as the beneficial therapy for PCOS. Docking analysis predicts the interaction between a ligand and a target protein, aiding drug discovery. The procedure entails the preparation of the protein and ligand, the identification of the binding site, and the optimization of the ligand. The docking algorithm produces many ligand conformations in the binding site, which are evaluated according to binding affinity. Ultimately, the highest-scoring poses are examined for critical interactions like as hydrogen bonds and hydrophobic contacts, yielding insights into binding mechanisms for additional confirmation [38]. A study performed on *Parquetina nigrescens * leaves identified 44 compounds from the extract, and the docking results revealed that the compound glutaric acid, 2-ethylbutyl heptyl ester (CID 91705405), showed the highest binding affinity to these proteins compared to standard drugs like spironolactone, clomiphene, and metformin. Specifically, CID 91705405 exhibited binding affinities of -8.4 Kcal/mol for AR, -8.1 Kcal/mol for ER, -8.0 Kcal/mol for FBP, and -8.1 Kcal/mol for PEPCK surpassing the affinities of reference drugs and they also had ideal RMSD and RMSF values making it more ideal and stable through molecular dynamics studies for a 100 ns run [39]. Similarly, a study on binding energies of the flaxseed-protein complexes was calculated, revealing values of -2.30 Kcal/mol for CCDC28b, -2.05 Kcal/mol for PDCD6IP, and -4.52 Kcal/mol for USP34, indicating stable interactions. These docked structures were further subjected to molecular dynamics (MD) simulations using the Galaxy platform with GROMACS tools to evaluate the stability and conformational changes of the protein-ligand complexes over time [40].

The study on isoquinoline alkaloids from the stem of *Tinospora cardifolia* revealed that the four alkaloids namely Palmatine (PAL), jatrorrhizine (JAT), magnoflorine (MAG), and berberine (BBR) were taken into the studies and docking was carried out by AutodockVina 4.2.6. and molecular dynamics by GROMACS. The findings emphasize that among the four constituents, two namely BER and PAL had an ideal binding energy of −8.23 Kcal/mol and −6.71 Kcal/mol and possessed a prominent complex stability in molecular dynamic environment against androgen receptors [41]. Our findings suggest that on analyzation of a series of 20 major phytoconstituents from the herbs, Sesamin from *Sesamum indicum* and Lanosterol from *Ficus religiosa* had high binding scores and interactions of −9.37 KJ/mol and −9.23 KJ/mol with androgen receptor and −9.63 KJ/mol and d −9.14 KJ/mol for progesterone receptor. Thus, the further experimental evaluation of these identified phytoconstituents is expected to yield promising results [42].

### 3.4 Pharmacokinetic and Toxicity Analysis

Determination of pharmacokinetic and toxicity parameters of the compounds in the early stages of Drug Discovery helps in saving a quantum of time. Various online tools and software available to perform the study are listed in Table **3**. The methodology typically starts with compound input, where the molecular structure or SMILES format is uploaded to the tool. ADME properties, such as solubility, permeability, and metabolic stability, are predicted based on computational models. A study conducted on vitamin E as a potential agent for PCOS explains that the compound showed ideal pharmacokinetic values within the range for parameters like absorption, metabolism, distribution, elimination, and toxicity performed by SWISS ADME and pkCSM online tools. The toxicity determination through OSIRIS property explorer explained that Vitamin E was non-toxic to all the four calculated parameters like mutagenicity, tumorigenicity, eye and skin irritation, and reproductive toxicity. Similarly, our findings involving 20 constituents provided data representing four compounds lanosterol, sesamin, alantolactone, and nimbolide possessed good drug-likeliness properties while sesamin and lanosterol were found to be safe and non-toxic through toxicity prediction [42, 43].

### 3.5 Statistical Analysis

The study of polycystic ovarian syndrome (PCOS) in bioinformatics relies on various statistical tools to analyze and interpret complex datasets. Principal component analysis (PCA), which summarises the variation in datasets, such as genetic or transcriptomic data, into principal components, is one often used technique that aids in reducing dimensionality and discovering patterns. Regression analysis, particularly logistic and linear regression, is another crucial technique for examining correlations between variables, such as the link between genetic variations and PCOS clinical features. Similar biological entities, such as genes or proteins, are grouped according to their expression profiles using hierarchical clustering and k-means clustering, which reveal underlying biochemical pathways [44, 45]. Machine learning algorithms, including support vector machines (SVM) and random forests, are increasingly applied to predict PCOS diagnosis and identify biomarkers by learning patterns from high-dimensional datasets. To identify genetic variations linked to PCOS, genome-wide association studies (GWAS) use statistical methods such as mixed-model analysis and the chi-square test [46]. Relationships between hormone levels, metabolic indices, and other clinical aspects are frequently investigated using correlation analyses, such as Pearson or Spearman correlation coefficients. Additionally, bioinformatics analysis uses survival analysis methods like Cox proportional hazards models and Kaplan-Meier curves to assess how different factors affect long-term outcomes in PCOS. By identifying gene-gene and protein-protein interactions, network analysis methods and statistical significance tests provide insights into the regulatory networks underlying PCOS [47]. When used properly, these statistical tools offer a strong framework for understanding the intricate biology and clinical features of PCOS, resulting in better diagnosis and treatment strategies.

## 4 EMERGING THERAPEUTIC APPROACHES

Historically, the management of PCOS has primarily focused on symptom control and the mitigation of associated risks, such as infertility and metabolic complications. Standard treatment regimens for PCOS typically include lifestyle modifications, hormonal contraceptives, insulin-sensitizing agents, and ovulation induction therapies. Although these approaches have been beneficial for many patients, they often do not provide comprehensive and long-term relief. Consequently, there is a growing demand for more effective and personalized therapeutic options. This chapter explores the most recent advances in therapeutic approaches for PCOS, emphasizing the latest research and clinical trials that are expected to enhance treatment efficacy and improve patient outcomes.

### 4.1 Genetic Applications of Gene Editing Technologies for PCOS

#### 4.1.1 Gene Correction

PCOS is characterized by a variety of symptoms, including disrupted follicular development and ovulation. Genetic variants, including those in FSHR, have been associated with this condition [48]. FSHR encodes the receptor for FSH, which is crucial for follicular growth and maturation; thus, mutations in this gene could potentially affect these processes. The correction of gene mutations and restoration of normal follicular development and ovulation through gene correction is a promising approach for the treatment of PCOS.

#### 4.1.2 Gene Knockout

Targeting and knocking out genes contributing to hyperandrogenism, or insulin resistance can mitigate key features of PCOS. Hyperandrogenism is central to the pathophysiology of PCOS, and insulin resistance plays a significant role in exacerbating clinical manifestations of the syndrome [49-51]. The interplay between hyperandrogenism and insulin resistance is complex, with evidence suggesting that hyperandrogenism may contribute to insulin resistance, and *vice versa* [52].

#### 4.1.3 Gene Activation/Repression

CRISPR activation (CRISPRa) and CRISPR interference (CRISPRi) are gene regulatory technologies that can modulate gene expression without altering the DNA sequence. CRISPRa facilitates the upregulation of gene expression, while CRISPRi is used to downregulate or silence genes [53, 54]. The application of CRISPRa and CRISPRi in the context of Polycystic Ovary Syndrome (PCOS) pathogenesis, can upregulate or downregulate the expression of specific genes involved in PCOS pathogenesis.

### 4.2 Targeted Drug Therapies Based on Molecular Pathways

PCOS involves various molecular pathways related to insulin signalling, androgen production, and inflammation. Understanding these pathways has led to the development of targeted drug therapies that address specific aspects of PCOS pathophysiology.

#### 4.2.1 Inositol and Other Nutraceuticals

Inositols, particularly myo-inositol (MI) and D-chiro-inositol (DCI), have garnered attention for their role in insulin signalling and ovarian function. Studies suggest that inositol supplementation can improve insulin sensitivity, reduce hyperandrogenism, and restore menstrual cyclicity in women with PCOS [55]. The combination of MI and DCI in a physiological ratio appears to offer the most benefit, enhancing ovulatory function and metabolic profiles.

Other nutraceuticals, such as N-acetylcysteine (NAC), omega-3 fatty acids, and vitamin D, also show promise in managing PCOS symptoms [56]. NAC, an antioxidant and insulin sensitizer, has been found to improve insulin resistance and ovulation rates. Omega-3 fatty acids, with their anti-inflammatory properties, may help reduce hyperandrogenism and improve lipid profiles [57]. Vitamin D supplementation, particularly in women with deficiency, can enhance insulin sensitivity and reproductive outcomes [58, 59]

#### 4.2.2 Gut Microbiota Modulation

Emerging evidence suggests a link between gut microbiota dysbiosis and PCOS pathogenesis. The gut microbiota influences metabolic homeostasis, inflammation, and androgen levels, all of which are relevant to PCOS [60, 61]. Probiotics, prebiotics, and synbiotics (a combination of both) are being explored to modulate the gut microbiota and improve metabolic and reproductive outcomes in PCOS patients [62, 63]. Clinical trials have demonstrated that specific probiotic strains can reduce body weight, insulin resistance, and androgen levels, highlighting the potential of gut microbiota modulation as a therapeutic strategy [64, 65]. The study of Zeng *et al. *[66] examines gut microbial dysbiosis in women with insulin-resistant PCOS (IR-PCOS), non-insulin-resistant PCOS (NIR-PCOS), and healthy controls. Using 16S rRNA gene sequencing, significant differences in gut microbial composition were identified among the groups. *Bacteroidaceae *abundance increased notably in IR-PCOS, correlating positively with insulin resistance, inflammation, and hormonal imbalances. In contrast, *Prevotellaceae,* known for its anti-inflammatory properties, was significantly reduced in PCOS patients, particularly in the IR-PCOS group, and negatively associated with inflammation and insulin resistance. Functional predictions revealed altered metabolic pathways in PCOS, with IR-PCOS showing increased pathways linked to lipopolysaccharide biosynthesis, exacerbating inflammation. Reduced alpha diversity and suppressed SCFA production were also observed, highlighting impaired gut function. These findings emphasize the distinct gut microbial profiles in IR-PCOS and NIR-PCOS and suggest targeting gut dysbiosis, particularly *Bacteroidaceae* and *Prevotellaceae*, for developing tailored treatments and drug strategies to mitigate inflammation and metabolic complications in PCOS patients.

#### 4.2.3 Anti-Müllerian Hormone (AMH) Modulation

AMH, produced by ovarian follicles, is elevated in women with PCOS and correlates with the severity of anovulation and hyperandrogenism [67]. Targeting AMH pathways is a novel approach being investigated for PCOS management [68]. AMH antagonists or inhibitors could potentially reduce follicular excess and restore normal ovarian function. While still in the early stages, this approach holds promise for addressing the ovarian dysfunction characteristic of PCOS [69].

#### 4.2.4 Advanced Reproductive Technologies

For women with PCOS seeking fertility, advanced reproductive technologies (ART) offer promising solutions [70]. Letrozole, an aromatase inhibitor, has emerged as a first-line ovulation induction agent, showing superior efficacy compared to clomiphene citrate. *in vitro* maturation (IVM) of oocytes, a technique that involves harvesting immature oocytes and maturing them in the laboratory, is particularly beneficial for PCOS patients at risk of ovarian hyperstimulation syndrome (OHSS) [71]. Additionally, personalized ART protocols, including tailored ovarian stimulation and embryo selection, are improving success rates and reducing complications.

#### 4.2.5 Anti-androgenic Therapies

Androgen receptor (AR) antagonists and 5-alpha-reductase inhibitors are being explored as more targeted therapies for hyperandrogenism in PCOS [72]. Spironolactone, an AR antagonist, and finasteride, a 5-alpha-reductase inhibitor, have shown efficacy in reducing hirsutism and acne. Novel agents with fewer side effects and higher specificity for androgen receptors are under investigation, offering the potential for improved management of androgenic symptoms in PCOS [73].

#### 4.2.6 Inflammatory Pathway Modulators

Chronic low-grade inflammation is implicated in PCOS pathogenesis [74]. Targeting inflammatory pathways with agents like anti-inflammatory cytokines or immune modulators can reduce inflammation and improve metabolic and reproductive outcomes [75, 76]. These therapies are in the early stages of development but hold promise for comprehensive management of PCOS [77, 78].

### 4.3 Regenerative Medicine and Stem Cell Therapy for Tissue Repair

#### 4.3.1 Stem Cell Therapy in PCOS

Regenerative medicine, specifically stem cell therapy, is posited as a promising avenue for addressing ovarian dysfunction in PCOS [79]. It underscores the potential of regenerative medicine, including stem cell therapy, as an alternative treatment for PCOS, given the limitations and risks associated with long-term pharmacological treatments and surgical options [80].

#### 4.3.2 Ovarian Tissue Regeneration

Stem cell therapy aims to regenerate ovarian tissue and restore normal folliculogenesis and hormone production. Mesenchymal stem cells (MSCs) and induced pluripotent stem cells (iPSCs) are being explored for their therapeutic potential in various medical conditions, including PCOS. The use of iPSCs derived from patients with PCOS to model the disease *in vitro* has been reported, providing a novel approach to studying the pathogenesis of PCOS and to screen for potential treatments [81, 82].

## 5 PREVENTION AND AWARENESS FOR PCOS

Preventative measures for PCOS are centered on lifestyle modifications, including diet, exercise, and behavioural changes, which are crucial along with maintaining a healthy weight to enhance insulin sensitivity and hormonal balance. These measures are not only essential for managing the condition but also for preventing the occurrence of associated complications. Regular medical check-ups are essential for early symptom detection and management to prevent complications.

Awareness efforts can be enhanced through educational programs such as workshops, seminars, and online resources, as well as support groups where individuals can share experiences and coping strategies. Several organizations have been dedicated to raising awareness about PCOS, such as the PCOS Awareness Association (https://www.pcosaa.org/), the PCOS Challenge: The National Polycystic Ovary Syndrome Association (https://pcoschallenge.org/) and the PCOS Society, India (pcosindia.org). Furthermore, public campaigns can significantly increase awareness and advocacy for better research and healthcare policies. Educational platforms like the PCOS Hub (The PCOS Hub | By Health & Balance Vitamins (handbvitamins.com)), the PCOS Collective (https://pcoscollective.com/) and also distributing educational materials such as pamphlets, brochures, articles, and podcasts and encouraging healthcare providers to undergo specialized training in PCOS management ensures that patients receive the best care based on updated clinical guidelines.

## 6 DISCUSSION

Research on polycystic ovary syndrome (PCOS) is critically important due to its high prevalence, affecting 8-13% of women worldwide, with even higher rates in certain regions like India. Effective and timely research is essential to develop better diagnostic and treatment options, improve fertility outcomes, and reduce the economic burden associated with managing PCOS and its complications. Thus, the utilization of recent innovations in research is to be done in an effective and precise manner. As discussed above, various innovations like trackers and applications are remotely available to all for self-diagnosis and monitoring of menstrual cycle patterns in women, ovulation streamlining and several algorithms assisting the practitioners in medical diagnosis of the disorder. These innovations should not be left unnoticed and utilized effectively.

A variety of computational methods, including online tools, web servers, and free software, are easily accessible for conducting bench research, significantly reducing both time and costs. Some discoveries, such as the role of single nucleotide polymorphisms (SNPs), particularly the CYP11A1 gene, have been identified as playing a key role in the pathophysiology of PCOS. Other studies have explored the impact of Staf overexpression and the involvement of E47 in regulating FSH, along with the role of transcription factor binding sites in the disorder's pathophysiology. Additionally, the results from network pharmacology offer significant insights into the connection between the AKT1 pathway and PCOS. The AKT1 pathway, a vital part of the PI3K/AKT signalling cascade, plays a significant role in folliculogenesis and ovarian function, while also mediating insulin's metabolic effects. Its dysregulation contributes to increased insulin resistance and androgen production. Therefore, further research into this specific pathway is anticipated to produce promising outcomes. Molecular docking, dynamics, and pharmacokinetic studies have significantly advanced the drug discovery process for Polycystic Ovarian Syndrome (PCOS) by enabling the design and evaluation of targeted therapeutics with higher precision and efficacy.

**Fig. (2):**
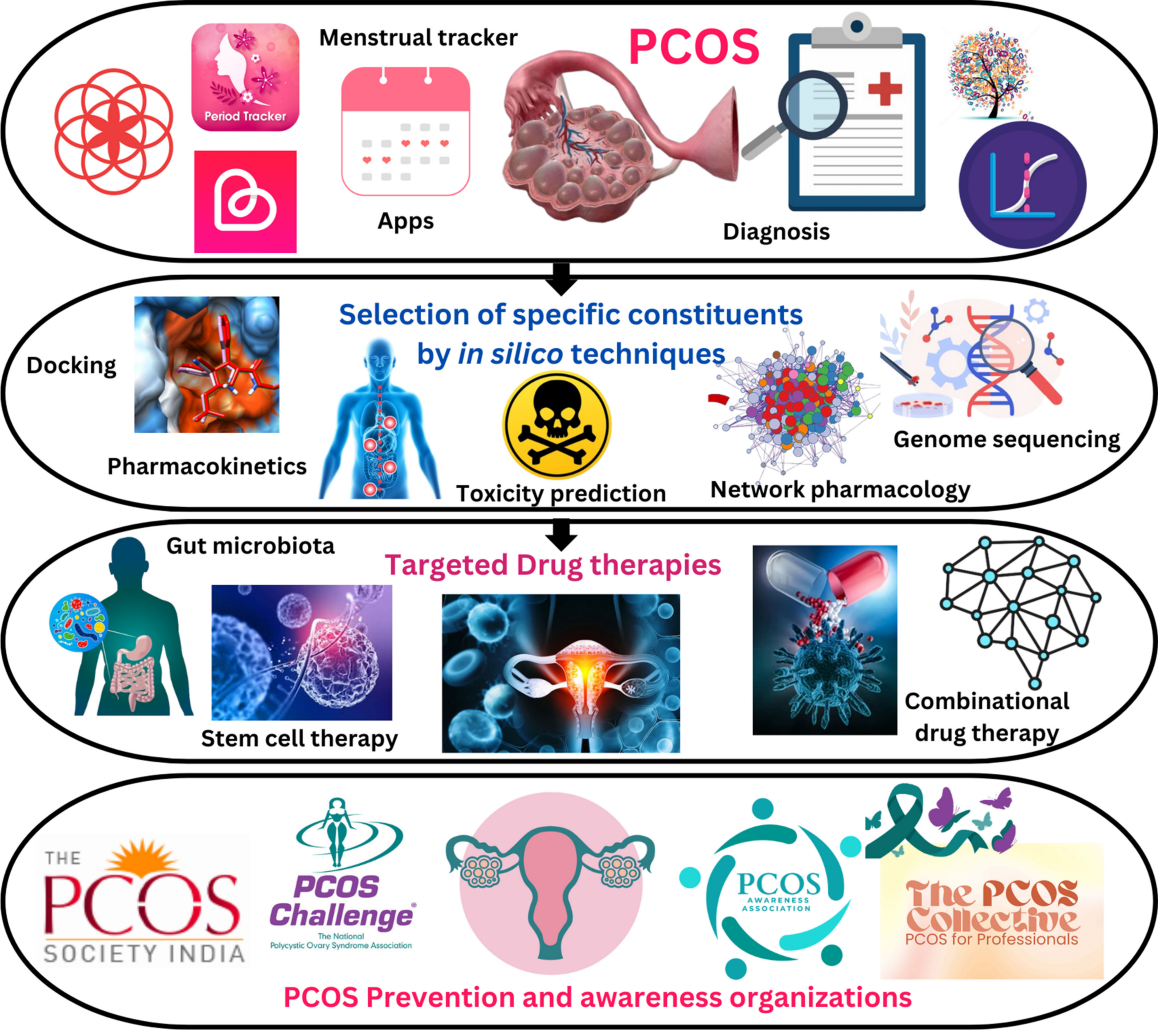
A complete workflow of recent innovations pertaining to the list of applications and techniques available for diagnosis of the disorder and several research strategies and methods used in targeting the symptoms for management of PCOS.

Molecular Docking facilitates the identification of potential drug candidates by predicting their binding affinity and interaction patterns with specific targets, such as androgen receptors, insulin receptors, or inflammatory mediators involved in PCOS. By modelling the interactions between ligands and receptor active sites, docking studies provide insights into the structural requirements for binding, helping in the rational design of compounds with enhanced specificity and potency. Molecular Dynamics (MD) simulations go a step further by assessing the stability and behaviour of drug-target complexes over time in a simulated biological environment. This aids in understanding the functional implications of drug binding and optimizing lead compounds to enhance their effectiveness under physiological conditions. Pharmacokinetic analysis complements the *in silico* approaches by predicting the absorption, distribution, metabolism, and excretion (ADME) profiles of potential drug candidates. Computational tools can evaluate properties like bioavailability, half-life, and potential toxicity, enabling the selection of compounds with favourable pharmacokinetic profiles. This reduces the risk of late-stage failures in drug development and accelerates the pipeline for bringing effective treatments to clinical trials. Together, these techniques enable a cost-effective and efficient exploration of novel therapeutic agents for PCOS, offering a pathway to develop drugs that target the underlying molecular mechanisms of the disorder with precision. By integrating docking, dynamics, and pharmacokinetics, researchers can design innovative solutions to address the multifactorial nature of PCOS, ultimately improving treatment outcomes and patient quality of life. The above findings from *in silico* docking and molecular dynamics techniques demonstrated that compounds such as Glutaric acid, 2-ethylbutyl heptyl ester, Palmatine, Berberine, Sesamin, and Lanosterol exhibit high binding affinity and strong complex stability without toxic effects, highlighting their potential in combating PCOS symptoms. Additionally, findings from various conventional targeted therapies underscore the effectiveness of MI and DCI combinations, the role of gut microbiota through probiotics and prebiotics, and the use of advanced reproductive technologies in addressing the symptoms and key pathways associated with PCOS [83]. These findings suggest that the integration of diagnostic applications and devices, followed by computational analysis of potential constituents targeting specific genes, and the application of emerging targeted therapy technologies, could lead to the development of potent treatments to combat PCOS symptoms (Fig. **2**).

## 7 LIMITATIONS

The review underscores the significance of innovative strategies in addressing polycystic ovary syndrome (PCOS) for the upcoming generation but acknowledges several limitations that must be addressed for future advancements. One of the primary challenges lies in the translation of next-generation technologies, such as genome-wide association studies (GWAS), transcriptomics, and computational approaches, into routine clinical practice. These technologies are often constrained by their high cost, limited accessibility, and the need for specialized expertise, which restricts their widespread adoption. Furthermore, while the integration of big data analytics and machine learning offers immense potential in PCOS management, these approaches require extensive validation to ensure their reliability and applicability across diverse populations and clinical settings. The promise of personalized medicine and novel pharmacological interventions is another highlight however, these approaches face unresolved issues regarding their long-term safety, efficacy, and scalability. Without robust evidence, these promising strategies cannot be universally applied. Moreover, the multifactorial nature of PCOS, which involves a complex interplay of genetic, environmental, and lifestyle factors, necessitates comprehensive longitudinal studies to deepen understanding. Unfortunately, this aspect remains insufficiently addressed in current explorations. These limitations underscore the critical need for multidisciplinary collaboration and sustained research efforts. By bridging the gap between innovation and real-world application, these endeavors can help translate cutting-edge strategies into accessible, effective, and sustainable solutions for management of PCOS.

## CONCLUSION

Thus, the overall information obtained through various studies emphasizes that the incorporation of advanced technologies and innovative strategies marks a transformative phase in PCOS research and management. Emerging approaches such as genome-wide association studies (GWAS), transcrip- tomics, and metabolomics are expected to unravel novel genetic and molecular markers, enabling earlier and more precise diagnosis. The integration of artificial intelligence and machine learning into big data analytics holds promise for developing predictive models that facilitate individualized treatment plans tailored to the complex pathophysiology of PCOS. Additionally, advancements in pharmacology and the identification of specific molecular targets could lead to therapies that address root causes, including inflammation, insulin resistance, and hormonal imbalances, rather than merely managing symptoms. The adoption of digital health tools, such as wearable devices and mobile applications, is anticipated to play a pivotal role in real-time patient monitoring, education, and self-management, potentially improving accessibility and equity in healthcare delivery. Furthermore, a holistic understanding of the interplay between genetic, environmental, and lifestyle factors is critical for achieving improved patient outcomes and enhancing the quality of life for those affected by PCOS. Over the next five years, a multidisciplinary approach combining next-generation research, technological innovations, and real-world applications will likely redefine the landscape of PCOS management. However, challenges such as ethical considerations, cost barriers, and the need for global standardization of protocols must be addressed. By critically analyzing and effectively applying these advancements, researchers and clinicians can drive significant progress, paving the way for more comprehensive, equitable, and effective care for PCOS patients. Challenges such as the lack of standardized diagnostic criteria, disparities in access to healthcare, and the limited representation of diverse populations in research must also be addressed. Bridging these gaps will require global collaboration and the development of cost-effective and scalable diagnostic and treatment protocols. By embracing a multidisciplinary approach that leverages technological advancements and emphasizes holistic patient care, future research can make significant strides in understanding and managing PCOS. Thus, this review will provide a comprehensive overview of how next-generation research is transforming the understanding, diagnosis, and treatment of polycystic diseases, supported by current advancements, technological innovations, and real-world applications, while also addressing the challenges and ethical considerations involved.

## Figures and Tables

**Table 1:** AI tools used for the diagnosis and monitoring of PCOS conditions.

**S. No.**	**PCOS Diagnostic Tools**	**Description**
**APPS**		
1	Clue	A menstrual and ovulation tracker app designed to monitor cycles, predict fertility windows, and log PCOS-specific symptoms such as irregular periods, anovulation, and hyperandrogenism.
2	Flo	A period tracker app that offers symptom tracking, personalized health insights, and reminders. Flo includes a specific PCOS management mode to help track related symptoms.
3	HealthifyMe	The HealthifyMe PCOS tracker app provides features and tools to help women manage and monitor their condition effectively.
4	My PCOS Tracker	Designed specifically for women with PCOS, this app tracks periods, symptoms, medications, and lifestyle changes, offering insights to identify potential PCOS indicators and support healthcare consultations.
5	Glow	A comprehensive health app that tracks menstrual cycles, ovulation, and symptoms. It includes community support and resources for women with PCOS.
6	Eve (by Glow)	Focuses on period tracking, symptom logging, and sexual health. It provides insights and community support for women dealing with PCOS.
7	Kindara	A fertility tracker app that helps monitor menstrual cycles, basal body temperature, and cervical fluid. It offers charting features that can be useful for women with PCOS.
8	Period Tracker (by GP Apps)	A simple app for tracking menstrual cycles, symptoms, and moods. It includes features for logging PCOS-specific symptoms.
**PCOS Tracker**		
9	Ovia Health	Ovia Fertility is particularly useful for tracking menstrual cycles, symptoms, and lifestyle factors, with a focus on women with PCOS.
10	Mira	An advanced fertility tracker that uses a smart analyzer and test wands to measure hormone levels. It provides personalized insights, which can be particularly useful for women with PCOS.
**AI and Machine Learning Models**		
11	Logistic Regression	A statistical model using a logistic function to analyze binary outcomes, commonly applied in classification tasks like medical diagnoses. It estimates probabilities of conditions such as PCOS, offering clear and interpretable results.
12	Support Vector Machines (SVM)	Support Vector Machines (SVMs) are supervised learning models used for classification and regression tasks. They are effective in distinguishing patients with and without PCOS based on clinical features and excel in handling high-dimensional, complex medical datasets.
13	Decision Trees	Easy to interpret and visualize, providing clear decision paths based on clinical features of PCOS like ultrasound findings and hormone levels.
14	Random Forests	Random forests are an ensemble learning method that constructs multiple decision trees during training and outputs the mode of the classes for classification tasks.
15	Neural Networks	Neural networks are a set of algorithms designed to imitate the functioning of the human brain in identifying patterns within a dataset. They are composed of input, hidden, and output layers.
16	Convolutional Neural Networks (CNNs)	Convolutional Neural Networks (CNNs) are a type of deep neural network primarily used for analyzing visual images. Effective in analyzing ultrasound images to identify polycystic ovaries and can be combined with clinical data for a more comprehensive diagnostic model.
17	Recurrent Neural Networks (RNNs)	RNNs are a type of neural network designed to recognize patterns in sequences of data such as time series or text. It is useful for analyzing time-series data, such as menstrual cycle patterns, and can capture temporal dependencies in patient data.
18	Gradient Boosting Machines (GBM) (Eg. XGBoost, LightGBM, and CatBoost)	GBMs are a family of machine learning algorithms that build models in a stage-wise fashion, optimizing for the best fit by minimizing loss functions. It has high predictive accuracy for structured data and is effective in handling heterogeneous data and feature interactions.

**Table 2:** *In silico* tools and servers aiding research related to gene identification and analysis for the management of PCOS symptoms.

**S.No**	**Name**	**Mode**	**Purpose**
**Gene Identification and Sequencing**			
1	GWAS catalogue (Genome-wide association studies)	Database	Identification of causative gene involved in the pathogenesis of PCOS.
2	ClueGO	Cytoscape plugin	Functional and biological interpretation of genes to constitute a network.
3	Ensembl	Genome browser	Genome browser comprising of vertebrae genomes.
4	PolyPhen 2	Online tool	Helps in the prediction of the impact of single amino acid substitution in the structure and function of human proteins.
5	SIFT (Sorting Intolerant from Tolerant)	Online tool	It analyzes sequence homology and the physical properties of amino acids to determine the potential effects of mutations on protein stability and function.
6	CADD (Combined Annotation-Dependent Depletion)	Online tool	To predict the deleteriousness of genetic variants by integrating multiple annotations into a single score, helping to identify variants that are likely to impact human health and disease.
7	Revel (Rare exome variant ensemble learner)	Website	To predict the pathogenicity of rare missense variants by combining scores from multiple computational tools into a single ensemble score and in identifying potentially disease-causing mutations in exome sequencing data.
8	Iterative Threading ASSEmbly Refinement (I-TASSER)	Online server	For protein structure prediction and function annotation.
9	MirSNP	Database	To analyze the impact of single-nucleotide polymorphisms (SNPs) in microRNA (miRNA) target sites, enabling researchers to understand how genetic variations influence miRNA binding, gene regulation, and disease susceptibility.
10	PolymiRTS	Database	In understanding the genetic basis of diseases and traits by exploring the impact of polymorphisms on gene regulation mediated by microRNAs.
11	miRNASNP 3	Online tool	It provides insights into how genetic variations can impact miRNA binding, expression, and regulatory networks, aiding in the study of genetic contributions to diseases and phenotypic traits.
12	SNP2TFBS	Web Interface	Analyze the impact of SNPs on TFBS
13	EnhancerDB	Database	Provide detailed information on enhancer elements in the human genome, aiding researchers in understanding gene regulation mechanisms.
14	HaploReg	Online tool	Explore the non-coding regions of the human genome, focusing on the regulatory potential of genetic variants.
15	PAINT (Promoter Analysis and Interaction Network Tool)	Computational tool	Analyze promoter regions and their interaction networks to identify transcription factor binding sites, uncover gene regulation mechanisms, and visualize interactions with regulatory proteins, supporting the study of gene expression and regulatory pathways.
16	TRANSFAC-Public	Database	Analyze transcription factor binding sites and their regulatory regions in the genome.
17	PHYRE2	Online server	Predicting the 3D structure of proteins based on their amino acid sequences. It employs advanced homology modelling techniques to identify structural templates and generate high-accuracy models.
18	CELLO2GO	Web server	Predict the sub-cellular localization and functional annotation of proteins by integrating protein sequence data with Gene Ontology (GO) terms, offering insights into cellular localization and potential biological roles.
19	ProtParam	Scientific database and online tool	Compute various physical and chemical properties of a protein sequence.
20	miRTarBase	Web server	It provides a comprehensive database of miRNA targets supported by strong experimental evidence.
21	DIANA-TarBase V8	Online tool	Exploring experimentally validated microRNA (miRNA) interactions with their target genes.
22	miEAA 2.0	Web server	Enrichment analysis of miRNA targets, providing functional insights and facilitating the understanding of miRNA roles in various biological processes and diseases.
23	Gene Expression Omnibus (GEO)	Public repository	Storing and accessing high-throughput gene expression and other functional genomics data.
24	HGDP-CEPH Human Genome Diversity Panel	Database and panel	For studying human genetic diversity, providing a resource for analyzing genetic variation and evolutionary history across different populations worldwide.
**Network Pharmacology**			
25	Cytoscape	Open-source software platform	Visualize and analyse molecular interaction networks and biological pathways by integrating diverse data types to construct complex network models, uncover relationships between genes and proteins, and identify key regulatory components in biological systems.
26	Cyto-Hubba plugin	Plugin	It provides various topological analysis methods to detect key nodes, such as bottlenecks and high-connectivity proteins, helping researchers understand critical components and interactions in complex biological systems.
27	PCOSKB	Database	Knowledge base specifically designed for polycystic ovary syndrome (PCOS). It compiles comprehensive information on genes, proteins, pathways, and other molecular entities associated with PCOS.
28	Uniprot	Database	Comprehensive resource for protein sequence and functional information. It provides detailed, curated data on protein sequences, structures, functions, and interactions.
29	STRING	Data resource	It combines established and predicted interactions from a variety of sources, such as experimental data, computational prediction techniques, and public text databases.
30	DrugBank	Database	Combines detailed drug data with drug target information. It provides information on drug interactions, mechanisms, chemical structures, pharmacology, and target proteins.
31	STITCH (Search Tool for Interacting Chemicals)	Database	It integrates data on chemical-protein interactions from various sources.
32	DAVID	Database	Functional annotation and enrichment analysis of gene lists.
33	DisGeNet	Web Interface	Integrates information on gene-disease associations from various sources, including scientific literature and curated databases.
34	GeneCard	Database	Detailed information on all annotated and predicted human genes.
35	Metascape	Web-based tool	For gene annotation and analysis, integrating various bioinformatics resources to provide comprehensive insights into gene function and biological pathways.
36	ChEMBL	Database of small compounds	It provides information on compound properties, targets, mechanisms of action, and pharmacology.

**Table 3:** *In silico* tools and servers aiding research related to medicinal chemistry aspects for the drug discovery process of PCOS.

**S.No**	**Name**	**Mode**	**Purpose**
**Molecular Docking**			
37	PubChem	Chemical Database	It provides comprehensive information on the chemical structures, properties, biological activities, safety, and toxicity of small molecules.
38	RCSB PDB	Repository	A repository for 3D structural data of biological macromolecules, including proteins and nucleic acids.
39	Autodock	Software	A suite of automated docking tools designed to predict how small molecules, such as substrates or drug candidates, bind to a receptor with a known 3D structure.
40	PyRx	Open-source virtual screening software	It integrates several docking programs, including AutoDock and AutoDock Vina, to facilitate the screening of large libraries of compounds against biological targets.
41	Swiss PDB viewer	Online tool	It provides tools for viewing, modelling, and refining protein structures.
42	Open babel	Tool/ software	Used to interconvert data
43	Biovia Discovery studio	Software	It provides advanced capabilities for molecular modelling, protein-ligand docking, pharmacophore modelling, and simulation, in visualizing, analyzing, and predicting the behaviour of biological and chemical systems.
44	Molegro molecular viewer	Software	Visualization tool used for analyzing molecular structures and docking results.
45	PyMol	Software	It is extensively utilized in structural biology, drug design, and computational chemistry for generating high-quality images and animations of molecular structures.
**Pharmacokinetics**			
46	pkCSM	Online tool	Predicting the pharmacokinetic and toxicity properties of small molecules. It integrates various computational models to assess ADMET properties.
47	admetSAR	Web interface	Predicting the ADMET characteristics of chemical compounds.
48	Swiss ADME	Web-based tool	Evaluating the pharmacokinetics, drug-likeness, and medicinal chemistry friendliness of small molecules.
49	Mol Inspiration	Web-based tool	It provides insights into various molecular descriptors and predicts the bioactivity of compounds against different biological targets.
**Toxicity Prediction**			
50	OSIRIS Property Explorer	Online tool	Evaluating the drug-likeness, toxicity, and overall safety profile of chemical compounds. It provides predictions for various properties such as mutagenicity, tumorigenicity, irritation, and reproductive effects.
51	CardioToxCSM	Web server	Predict the cardiotoxicity of small molecules. It leverages computational models to assess the potential for a compound to cause adverse effects on the cardiovascular system.
52	embryoTox	Online tool	Predict the embryotoxicity of chemical compounds, assessing their potential to cause adverse effects on embryonic development.
53	Pro Tox II	Webserver	Predict the toxicity of chemical compounds, including acute toxicity, hepatotoxicity, cytotoxicity, and other toxicological endpoints.
**Biological Activity Prediction**			
54	PASS (Prediction of Activity Spectra for Substances) online	Online tool	Predict the biological activity spectrum of chemical compounds. It employs computational models to assess the potential pharmacological effects, mechanisms of action, and possible side effects of compounds based on their chemical structure.
**Molecular Dynamics and Free Energy Calculations**			
55	GROMACS	Software	Molecular dynamics simulation software used for simulating the motion of atoms and molecules.
56	Schrodinger suite	Software	It provides tools for molecular visualization, structure-based drug design, molecular dynamics simulations, quantum mechanics calculations, and cheminformatics.

## LIST OF ABBREVIATIONS

SHBG: Sex Hormone-binding Globulin
LH: Luteinizing Hormone
GWAS: Genome-wide Association Studies
NGS: Next-generation Sequencing
PCOS: Polycystic Ovary Syndrome
